# Defining Multidrug Resistance of Gram-Negative Bacteria in the Dutch–German Border Region—Impact of National Guidelines

**DOI:** 10.3390/microorganisms6010011

**Published:** 2018-01-26

**Authors:** Robin Köck, Philipp Siemer, Jutta Esser, Stefanie Kampmeier, Matthijs S. Berends, Corinna Glasner, Jan P. Arends, Karsten Becker, Alexander W. Friedrich

**Affiliations:** 1 Institute of Hospital Hygiene Oldenburg, 26133 Oldenburg, Germany; 2 Institute of Hygiene, University Hospital Münster, 48149 Münster, Germany; stefanie.kampmeier@ukmuenster.de; 3 Institute of Medical Microbiology, University Hospital Münster, 48149 Münster, Germany; kbecker@uni-muenster.de; 4 European Medical School Oldenburg-Groningen, 26129 Oldenburg, Germany; philipp.siemer@uni-oldenburg.de; 5 Laborarztpraxis Osnabrück, 49124 Georgsmarienhütte, Germany; j.esser@oslab.de; 6 Certe Medical Diagnostics & Advice, 9700 AX Groningen, The Netherlands; m.berends@certe.nl; 7 University of Groningen, University Medical Center Groningen (UMCG), Department of Medical Microbiology, 9713 GZ Groningen, The Netherlands; c.glasner@umcg.nl (C.G.); j.p.arends@umcg.nl (J.P.A.); alex.friedrich@umcg.nl (A.W.F.)

**Keywords:** ESBL, carbapenemase, Euregio, multidrug resistance, *Klebsiella*, *Escherichia coli*, *Acinetobacter baumannii*, *Stenotrophomonas maltophilia*, *Enterobacteriaceae*

## Abstract

Preventing the spread of multidrug-resistant Gram-negative bacteria (MDRGNB) is a public health priority. However, the definition of MDRGNB applied for planning infection prevention measures such as barrier precautions differs depending on national guidelines. This is particularly relevant in the Dutch–German border region, where patients are transferred between healthcare facilities located in the two different countries, because clinicians and infection control personnel must understand antibiograms indicating MDRGNB from both sides of the border and using both national guidelines. This retrospective study aimed to compare antibiograms of Gram-negative bacteria and classify them using the Dutch and German national standards for MDRGNB definition. A total of 31,787 antibiograms from six Dutch and four German hospitals were classified. Overall, 73.7% were no MDRGNB according to both guidelines. According to the Dutch and German guideline, 7772/31,787 (24.5%) and 4586/31,787 (12.9%) were MDRGNB, respectively (*p* < 0.0001). Major divergent classifications were observed for extended-spectrum β-lactamase (ESBL) -producing *Enterobacteriaceae*, non-carbapenemase-producing carbapenem-resistant *Enterobacteriaceae*, *Pseudomonas aeruginosa* and *Stenotrophomonas maltophilia*. The observed differences show that medical staff must carefully check previous diagnostic findings when patients are transferred across the Dutch–German border, as it cannot be assumed that MDRGNB requiring special hygiene precautions are marked in the transferred antibiograms in accordance with both national guidelines.

## 1. Introduction

Antimicrobial multidrug-resistant Gram-negative bacteria (MDRGNB) globally challenge clinicians and infection control personnel due to limited treatment options and the need to implement barrier precautions for preventing MDRGNB transmission [1]. Comparing this challenge is particularly interesting in neighboring regions characterized by highly developed but structurally different healthcare systems. An example for such a region is the Dutch–German border area, which is inhabited by 12 million people and comprises >100 hospitals.

In The Netherlands and Germany, surveillance systems currently indicate that 7.0% and 11.8% of all *Escherichia coli* and 10.8% and 14.3% of all *Klebsiella pneumoniae* isolated from blood cultures are non-susceptible to third-generation-cephalosporins indicative for production of extended-spectrum β-lactamases (ESBL) [2]. Moreover, carbapenemase-producing *Enterobacteriaceae* (CPE) occur in both countries, although the overall meropenem or imipenem resistance rates of *Enterobacteriaceae* (e.g., *Klebsiella* spp.) are still <1% [2]. Thirdly, carbapenem resistance in *Acinetobacter baumannii*, which is often due to carbapenem-hydrolysing oxacillinase (OXA) production, affects 1.9% and 5.4% of all invasive isolates in The Netherlands and Germany respectively [2]. A fourth clinically relevant species is *Pseudomonas aeruginosa*. For this bacterium, 11% and 18% of all isolates from bloodstream infections were non-susceptible to ceftazidime and meropenem in Germany, respectively. In contrast, resistance rates were 3.5% and 6.1% in The Netherlands [2].

Nosocomial transmission is a major reason why the incidence of MDRGNB increases. Hence, infection control guidelines describing measures to prevent MDRGNB dissemination are implemented in many countries including The Netherlands and Germany. However, it should be noted that, according to data from the European Centre for Disease Prevention and Control (ECDC), Germany is currently considered as a country, where CPE are regionally endemic indicating inter-institutional spread, while their occurrence is more limited in The Netherlands. The same is observed for carbapenem-resistant *A. baumannii* [3]. This highlights the need to critically evaluate and compare infection control guidelines, as well as different risks for MDRGNB spread in these two countries. 

In this context, one aspect is the definition of MDRGNB. Although definitions for multidrug resistance in epidemiological studies are available [4], and although theoretically CPE or ESBL-producing *Enterobacteriaceae* are clearly defined by harboring respective resistance encoding genes, the questions concerning what MDRGNB are in clinical routine and for which MDRGNB special barrier precautions should be implemented, are not universally defined. Moreover, it is important to differentiate between MDRGNB definitions established for therapeutic decisions and those created for epidemiological purposes and infection prevention [4]. Recently, Mueller et al. have pointed out differences between the Dutch and German guidelines regarding the advice they give to laboratories and infection control personnel, which Gram-negative bacteria and antimicrobial resistance patterns should be considered as MDRGNB [5]. As patient movement across the Dutch–German border is not infrequent, such divergent definitions could result in reduced patient safety, because isolates requiring isolation in the hospital abroad are not flagged as being multidrug-resistant on the microbiological reports.

Hence, in this article, we collected antibiograms of Gram-negative bacteria from Dutch and German hospitals located in the border region and applied both national MDRGNB definitions for infection prevention measures on this dataset. The results of this comparison shall clarify the differences between the two countries and estimate the impact of these differences for daily infection control practice.

## 2. Materials and Methods 

We retrospectively extracted antibiograms of Gram-negative bacteria from laboratory information systems. Data about phenotypic and genotypic ESBL and carbapenemase tests performed for the respective bacterial isolates were also extracted, if available. All isolates included originate from patients treated in six Dutch and four German hospitals. All hospitals are located in the Dutch–German border region including the Northern part (Ems Dollart region) and the central part (EUREGIO). Five of six Dutch hospitals provided datasets from 1 January 2015 to 31 December 2016, because only a small number of samples was tested in these facilities; the sixth Dutch hospital and the German hospitals provided data for 2016 only. Antimicrobial susceptibility testing was done using guidelines of the European Committee on Antimicrobial Susceptibility Testing (EUCAST) guidelines and clinical breakpoints in all laboratories.

Anonymization of patient-related and hospital-related data was done before analysis. We initially included all Gram-negative bacterial species and then restricted the dataset to *Enterobacteriaceae*, *P. aeruginosa*, *A. baumannii* complex, and *Stenotrophomonas maltophilia*, as these are the species for which recommendations regarding MDRGNB definitions and special hygiene precautions are included in Dutch and German infection control guidelines [6,7]. We included all isolates; duplicate isolates from the same patient were not removed. Classification of MDRGNB was done according the German national guideline (MDRGNB classified according to this guideline are henceforth designated “Multiresistente Gramnegative Stäbchen”, MRGN, with the subtypes 3MRGN and 4MRGN) summarized in Table 1 [6] and according to the Dutch national guideline (MDRGNB according to this guideline are henceforth designated “Bijzonder Resistente Micro-Organismen”, BRMO) shown in Table 2 [7], for all isolates including complete phenotypic susceptibility test data for the antibiotics mentioned. Incomplete antibiograms were deleted from the dataset. 

Statistical analysis was done by EpiInfo (version. 7.0, CDC Atlanta, Atlanta, GA, USA) using Chi-Square or (where appropriate) Fisher’s exact test; *p* < 0.05 was considered significant. The final dataset does not allow for conclusions about the epidemiology or the prevalence of MDRGNB, as it contains both isolates obtained from screening asymptomatic patients and clinical specimens. Moreover, the diagnostic procedures and indications for screening and clinical diagnostics were not harmonized in the participating laboratories and hospitals.

## 3. Results

### 3.1. Number of Antibiograms and Patients

Initially, 35,619 antibiograms were included of which 12,616 were from Dutch and 23,003 from German hospitals. The 12,616 isolates were from five Dutch secondary-care hospitals (*n* = 4377; from 2015 to 2016) and one Dutch university-hospital (*n* = 8239, 2016), and the 23,003 isolates were from three German secondary-care hospitals (*n* = 6914, 2016) and one German university-hospital (*n* = 16,089, 2016). Overall, 80.9% of all isolates were *Enterobacteriaceae* and 19.1% non-fermenting Gram-negative bacteria. 

When analyzing the data, two major limitations occurred: (i) For *Enterobacteriaceae*, the Dutch classification system could not be applied for 3832 isolates, because they were not tested for the presence of ESBLs or test results were unclear (*n* = 3720 isolates from the German hospitals and *n* = 112 from Dutch hospitals). This is because testing for the presence of ESBL is not required by the German MRGN classification system (and is often not performed in German laboratories except for *E. coli* and *Klebsiella* spp., where this test is routinely implemented in automated systems used for antimicrobial susceptibility testing). These isolates were therefore excluded from further analysis, which reduced the total number of isolates analyzed to 31,787. (ii) Overall, we lacked data for the results of carbapenemase PCRs for non-fermenting bacteria. As carbapenemase PCRs are not required for classification in the German system, these results were not available for 4651 *P. aeruginosa* isolates from German hospitals. Since no VIM-carbapenemase was reported for the 1205 *P. aeruginosa* isolates from Dutch hospitals, we coped with this problem by assuming that the German *P. aeruginosa* isolates were also VIM-negative and classified these isolates accordingly when applying the Dutch guideline. In contrast, for *A. baumannii*, we considered all carbapenem-resistant isolates as carbapenemase producers when applying the Dutch guidelines. For *Enterobacteriaceae*, test results were available, because German laboratories test the isolates in line with quality management measures.

### 3.2. Results of MRGN and BRMO Classification

According to the Dutch classification system, 7,772/31,787 (24.5%) isolates were BRMO. Applying the German classification system on the same antibiograms resulted in the identification of 4586/31l,787 (12.9%) MRGN (*p* < 0.0001). Table 3 shows where the two classification systems had the most divergent results on genus or species level.

**Figure 1:**
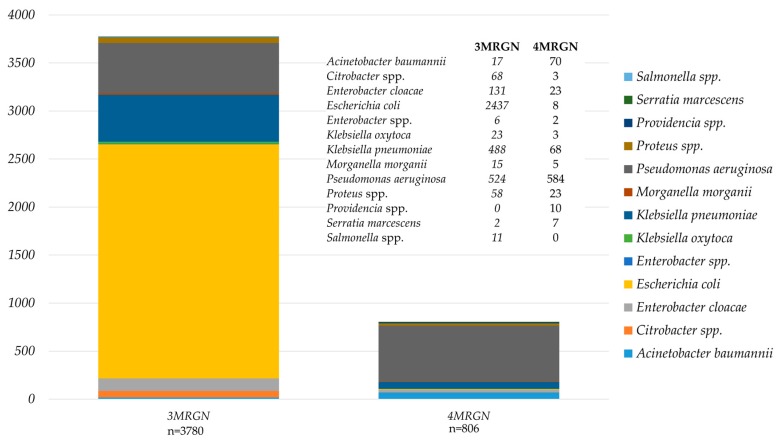
Species distribution among isolates classified as 3MRGN and 4MRGN according to the German guideline.

The distribution of 3MRGN and 4MRGN among the 4,586 MRGN isolates is shown in Figure 1. Among all 152 carbapenem-resistant *Enterobacteriaceae* isolates, carbapenemases were detected in 42 isolates (27.6%) with OXA-48-like genes being predominant. The remaining 110 isolates, were negative for carbapenemases (*n* = 87, 79.1%) or not tested (*n* = 23, 20.9%) and were meropenem-non-susceptible *Morganella*, *Proteus*, *Providencia*, and *Serratia* (*n* = 45), as well as *Klebsiella* spp. (*n* = 31), *Enterobacter* (*n* = 24), *E. coli* (*n* = 7), and *Citrobacter* (*n* = 3).

Of all 6882 isolates classified as BRMO-*Enterobacteriaceae*, 34 harbored carbapenemase-encoding genes (0.5%), 4953 were ESBL-producers (80.0%), and 3037 (44.1%) isolates were simultaneously resistant to fluoroquinolones and aminoglycosides. A total 788 *P. aeruginosa* isolates were classified as BRMO, because they had a resistance pattern in accordance with Table 2. Among the remaining 5058 *P. aeruginosa* isolates (3961 from German and 1107 from Dutch laboratories) not classified as BRMO, 1107 (21.9%) were carbapenem-resistant (981 and 126 from German and Dutch laboratories, respectively). A total 72 BRMO-*A. baumannii* isolates were classified as such, because they were carbapenem-resistant (*n* = 70), quinolone/aminoglycoside-resistant (*n* = 2) or both (*n* = 59). 

However, of all isolates 23,433 (73.7%) were neither classified as MRGN, nor as BRMO. Among 3780 and 806 isolates classified as 3MRGN and 4MRGN according to the German guideline, 3271 (86.5%) and 733 (90.9%) were also classified as BRMO. In contrast, of the 7772 isolates classified as BRMO, 3768 were not classified MRGN (48.5%). An agreement matrix between the Dutch and German guidelines for MDRGNB classification is shown in Table 4. 

## 4. Discussion

When patients are transferred between hospitals, information regarding MDRGNB colonization or infection must also be transferred to ensure continuous implementation of infection control measures. This is usually supported by indicating on antibiograms, which are included in the records of a transferred patient, whether the respective bacteria are multidrug-resistant according to the national guideline. For cross-border healthcare, this implies that clinicians or infection control staff can interpret antibiograms according to guidelines from both countries or understand foreign ‘MDRGNB languages’. The aim of this study was to describe different classifications used in The Netherlands and Germany in order to estimate the risk, which might be caused when patients infected or colonized with MDRGNB are transferred across the border without recognizing the respective bacteria as multidrug-resistant.

When planning the data analysis, a first hurdle occurred when the authors tried to actually understand the respective classification guidelines in detail. We learned that parts of the practical applicability of the guidelines (from both sides of the border) are rather locally defined. For example, in the Dutch guideline, it is not explicitly mentioned for *Enterobacteriaceae*, which fluoroquinolones (e.g., ciprofloxacin, levofloxacin, norfloxacin, moxifloxacin) and aminoglycosides (e.g., gentamicin, tobramycin, amikacin) should be considered for the classification of which bacterial species and how to categorize, if one quinolone is resistant and the other susceptible. In the German guideline, some exceptional rules, such as ignoring imipenem non-susceptibility in *Serratia* or *Proteus* for the classification (due to unreliable test results) are not mentioned, and can only be concluded from other guidance papers or publications of German reference laboratories. This might cause problems if microbiological laboratories are working across the border and might be perceived as a lack of transparency. This issue could be improved when national policy makers published more detailed standard operating procedures for laboratories where the problems occurring in daily routine are more accurately described.

Overall, the Dutch guideline makes it more laborious for a microbiological laboratory to actually classify an isolate as MDRGNB (tests for phenotypic ESBL-production and VIM-carbapenemase encoding genes). This might reflect structural differences in the organization of microbiological diagnostics between the two countries, as more laborious confirmation testing requires using more financial resources.

When comparing the Dutch and the German classification systems for MDRGNB (Table 4), we found very divergent results.

The bulk of isolates, which were classified differently, were *E. coli* and *Klebsiella* isolates characterized by ESBL-production, but being susceptible to fluoroquinolones. In German hospitals, no other than basic hygiene measures are taken for patients colonized or infected with these strains. This can be criticized, because spread of ESBL-producers might increase carbapenem use. Moreover, ESBLs are usually encoded on plasmids, which can be transferred independently from the bacterial clone even to other bacterial species. However, recent investigations have shown, that clonal spread of ESBL-*E. coli* in healthcare settings is rarely observed [8,9]. A second reason for divergent classifications was that the Dutch guideline uses combined fluoroquinolone and aminoglycoside resistance as a criterion for multidrug resistance. Aminoglycosides are not considered in the German guideline, maybe due to their limited and decreasing use in German hospitals compared with The Netherlands (<0.5 vs. 3.7 daily defined doses (DDD)/100 patient-days) [10,11]. Thirdly, major differences were also found for *P. aeruginosa*. Many of the very broadly resistant *P. aeruginosa* isolates, for which colistin, tobramycin, or new β-lactams (such as ceftolozane/tazobactam) were the only remaining treatment options, were not classified as MDRGNB by the Dutch guideline, because VIM-carbapenemases were not detected. In this context, we clearly overestimated the disagreement between the Dutch and German guideline (Table 4), because we considered all 1,107 carbapenem-resistant *P. aeruginosa* isolates (of 5058 not classified as BRMO) as VIM-negative. This is not probable as it is well known that in Germany up to 24% of carbapenem-resistant *P. aeruginosa* isolates harbor carbapenemases among which VIM is predominant [12]. This points towards a major limitation of this study. Since the analysis was not prospectively planned, we had to cope with missing data. Of course, excluding 3832 antibiograms (which is >10% of the antibiograms collected in the participating hospitals) due to a lack of information about phenotypic ESBL-test results for non-*E. coli*/non-*Klebsiella* isolates might have caused significant impact on the results. However, the total numbers *Enterobacter*, *Citrobacter*, or *Hafnia* isolates included from both sides of the border was comparable. Overall, the results of this study demonstrate that in contrast to other multidrug-resistant bacteria such as methicillin-resistant *Staphylococcus aureus* or vancomycin-resistant enterococci, those resistance pheno- or genotypes that define Gram-negative bacteria as MDRGNB markedly differ between The Netherlands and Germany. For cross-border care, the easiest solution would be to harmonize the classification rules of both countries. As long as this is not done, the full antibiogram data of Gram-negative bacteria should be transferred together with the patient in order to enable classification by local infection control staff. 

## Figures and Tables

**Table 1:** Classification according to German guideline into 3MRGN and 4MRGN.

Bacteria	Categories ^1^				Classification	
	I	II	III	IV	3MRGN ^5^	4MRGN ^5^
*Enterobacteriaceae* ^2^	PIP	TAX	CIP	IMI ^3^ or MER or CARB	Resistance to three of four categories	Resistance to four of four categories or to category IV alone
*P. aeruginosa*	PIP	TAZ and FEM	CIP	IMI and MER	Resistance to three of four categories	Resistance to four of four categories
*A. baumannii*	PIP ^4^	TAZ ^4^	CIP	IMI or MER or CARB	Resistance to three of four categories	Resistance to four of four categories or to category IV alone

^1^ PIP = piperacillin, TAX = cefotaxime, TAZ = ceftazidime, FEM = cefepime, CIP = ciprofloxacin, IMI = imipenem, MER = meropenem, CARB = carbapenemase detected in the isolate irrespectively of the resistance phenotype, intermediate test results are considered as resistant for the classification. ^2^ *Enterobacteriaceae* includes a classification for the following species: *E. coli*, *Klebsiella* spp., *Proteus* spp., *Citrobacter* spp., *Enterobacter* spp. and *E. cloacae*, *S. marcescens*, *Morganella* spp., *Providencia* spp. ^3^ Imipenem is not considered for *Proteus* spp., *Morganella* spp., *S. marcescens*, *Providencia* spp. ^4^ PIP and TAZ are always considered as resistant due to missing clinical breakpoints for *A. baumannii*. ^5^ Special hygiene precautions are required for patients with 4MRGN in all parts of the hospital and for patients with 3MRGN only on intensive care units or other units with immunocompromised patients according to local risk assessments.

**Table 2:** Classification according to Dutch guideline into BRMO.

Bacteria	Categories ^1^						
	ESBL	CARB ^1^	FQ	AM	PIP	TAZ	SXT
*Enterobacteriaceae* ^2^	BRMO ^3^	BRMO	BRMO: Resistance to FQ and AM ^2^		-		-
*P. aeruginosa*	-	BRMO: Resistance to ≥3 categories: CARB ^1^, FQ; AM; PIP; TAZ					-
*A. baumannii*	-	BRMO	BRMO: Resistance to FQ and AM ^2^		-		-
*S. maltophilia*	-	*-*	-				BRMO

^1^ CARB = carbapenemase, for *Enterobacteriaceae* at least OXA-48, Verona integron-encoded metallo-β-lactamase (VIM), New Delhi metallo-β-lactamase (NDM), *Klebsiella pneumoniae* carbapenemase (KPC), imipenem-carbapenemase (IMP) are tested; for *P. aeruginosa* at least VIM is tested, FQ = fluoroquinolones, AM = aminoglycosides, PIP = piperacillin, TAZ = ceftazidime. ^2^ FQ includes ciprofloxacin (and levofloxacin for *A. baumannii*); AM includes gentamicin and tobramycin (if tested both, resistance was assumed, if both were resistant. If only one of these agents was tested, this result was used for classification). ^3^ Classification as BRMO is followed by isolation in single-rooms (with anteroom) and barrier precautions for all *A. baumannii* and all *Enterobacteriaceae* characterized by CARB. For all other BRMO, contact isolation is recommended and can be done in single rooms or other rooms.

**Table 3:** Differences in using Dutch and German multidrug resistance classification systems.

	Dutch Classification		German Classification		
	BRMO ^1^	no BRMO ^1^	MRGN ^1^	no MRGN ^1^	*p*
*A. baumannii*	72	370	87	355	0.2202
*Citrobacter* spp.	79	626	71	634	0.5454
*E. cloacae*	146	972	154	964	0.6641
*E. coli*	5270	9606	2445	12,431	<0.0001
*Enterobacter* spp.	8	280	8	280	1
*Hafnia* spp.	1	44	0	45	1
*K. oxytoca*	75	1885	26	1934	0.0001
*K. pneumoniae*	877	2578	556	2899	<0.0001
*Klebsiella* spp.	0	17	0	17	1
*Morganella* spp.	45	226	20	251	0.0015
*P. aeruginosa*	788	5068	1108	4748	<0.0001
*Proteus* spp.	257	1009	81	1185	<0.0001
*Providencia* spp.	16	33	10	39	0.2526
*S. maltophilia*	30	471	0	501	<0.0001
*Salmonella*	95	17	11	101	<0.0001
*S. marcescens*	11	770	9	772	0.8219
*Serratia* spp.	2	43	0	45	0.4944
Total	7772	24,015	4586	27,201	<0.0001

^1^ BRMO = “Bijzonder Resistente Micro-Organismen”, according to Dutch classification system; MRGN = “Multiresistente gramnegative Stäbchen”, according to German classification system. *p* < 0.05 in bold.

**Table 4:** Correlation matrix between the Dutch BRMO-classification and the German MRGN-classification system to define multidrug-resistant Gram-negative bacteria (MDRGNB) for 31,787 isolates of different bacterial species

	MRGN % BRMO ^1^	MRGN % BRMO/CARB ^2^	BRMO % 3MRGN ^3^	BRMO % 4MRGN ^3^	BRMO % MRGN (All) ^3^	no MDRGNB ^4^	no Isolates ^5^
*Acinetobacter baumannii*	98.6	100.0	5.9	100.0	81.6	80.1	442
*Citrobacter* spp.	54.4	100.0	58.8	100.0	60.6	84.8	705
*Enterobacter cloacae*	58.2	100.0	57.3	43.5	55.2	80.8	1118
*Enterobacter* spp.	12.5	100.0	16.7	0.0	12.5	94.8	288
*Escherichia coli*	45.5	100.0	98.3	37.5	98.1	63.3	14,876
*Hafnia* spp.	0.0	100.0	100.0	100.0	100.0	97.8	45
*Klebsiella oxytoca*	21.3	100.0	60.9	66.7	61.5	95.7	1960
*Klebsiella pneumoniae*	61.7	100.0	99.0	85.3	97.3	74.2	3455
*Klebsiella* spp.	100.0	100.0	100.0	100.0	100.0	100.0	17
*Morganella* spp.	33.3	100.0	67.0	100.0	75.0	81.5	271
*Proteus* spp.	30.0	100.0	96.6	91.3	95.1	79.4	1266
*Providencia* spp.	62.5	100.0	100.0	100.0	100.0	67.3	49
*Pseudomonas aeruginosa*	92.8	100.0	34.7	94.0	66.0	80.1	5856
*Salmonella* spp.	11.6	100.0	100.0	100.0	100.0	15.2	112
*Serratia marcescens*	36.4	100.0	100.0	28.6	44.4	98.0	781
*Serratia* spp.	0.0	100.0	100.0	100.0	100.0	95.6	45
*Stenotrophomonas maltophilia*	0.0	100.0	100.0	100.0	100.0	94.0	501
Legend	≥75.0%			25.0–74.9%		<25.0%	

^1^ Percentage of BRMO also classified as MRGN (including 3MRGN and 4MRGN). ^2^ Percentage of carbapenemase-producing BRMO also classified as 4MRGN. ^3^ Percentage of 3MRGN, 4MRGN, and all MRGN also classified as BRMO. ^4^ Percentage of isolates not classified as MDRGNB by both the Dutch and German definitions. ^5^ Number of isolates included for the respective species.

## References

[1] Magiorakos A.P., Burns K., Rodríguez Baño J., Borg M., Daikos G., Dumpis U., Lucet J.C., Moro M.L., Tacconelli E., Simonsen G.S. (2017). Infection prevention and control measures and tools for the prevention of entry of carbapenem-resistant *Enterobacteriaceae* into healthcare settings: Guidance from the European Centre for Disease Prevention and Control. Antimicrob. Resist. Infect. Control.

[2] European Centre for Disease Prevention and Control EARS-Net Resistance Data for 2016. http://atlas.ecdc.europa.eu/public/index.aspx.

[3] Albiger B., Glasner C., Struelens M.J., Grundmann H., Monnet D.L. (2015). European Survey of Carbapenemase-Producing *Enterobacteriaceae* (EuSCAPE) Working Group. Carbapenemase-producing *Enterobacteriaceae* in Europe: Assessment by national experts from 38 countries, May 2015. Euro Surveill..

[4] Magiorakos A.-P., Srinivasan A., Carey R.B., Carmeli Y., Falagas M.E., Giske C.G., Harbarth S., Hindler J.F., Kahlmeter G., Olsson-Liljequist B. (2012). Multidrug-resistant, extensively drug-resistant and pandrug-resistant bacteria: An international expert proposal for interim standard definitions for acquired resistance. Clin. Microbiol. Infect..

[5] Müller J., Voss A., Köck R., Sinha B., Rossen J.W., Kaase M., Mielke M., Daniels-Haardt I., Jurke A., Hendrix R. (2015). Cross-border comparison of the Dutch and German guidelines on multidrug-resistant Gram-negative microorganisms. Antimicrob. Resist. Infect. Control.

[6] Kommission für Krankenhaushygiene und Infektionsprävention (KRINKO) (2012). Hygienemaßnahmen bei Infektionen oder Besiedlung mit multiresistenten gramnegativen Stäbchen. Bundesgesundheitsbl.

[7] Werkgroep Infectiepreventie BRMO (Bijzonder Resistente Micro-Organismen)—Ziekenhuizen. http://www.rivm.nl/dsresource?objectid=b6b99580-44e2-4b9c-8183-52871e61764f&type=org&disposition=inline.

[8] Mellmann A., Bletz S., Böking T., Kipp F., Becker K., Schultes A., Prior K., Harmsen D. (2016). Real-Time Genome Sequencing of Resistant Bacteria Provides Precision Infection Control in an Institutional Setting. J. Clin. Microbiol..

[9] Kluytmans-van den Bergh M.F.Q., van Mens S.P., Haverkate M.R., Bootsma M.C.J., Kluytmans J.A.J.W., Bonten M.J.M., SoM Study Group (2018). Quantifying Hospital-Acquired Carriage of Extended-Spectrum Beta-Lactamase-Producing Enterobacteriaceae among Patients in Dutch Hospitals. Infect. Control Hosp. Epidemiol..

[10] SWAB NethMap 2017—Consumption of Antimicrobial Agents and Antimicrobial Resistance among Medically Important Bacteria in The Netherlands in 2016. http://www.swab.nl.

[11] Paul-Ehrlich Gesellschaft (PEG) Bundesamt für Verbraucherschutz und Lebensmittelsicherheit, GERMAP 2015—Antibiotika-Resistenz und –Verbrauch. http://www.p-e-g.org/econtext/germap.

[12] Kaase M. (2016). Bericht des Nationalen Referenzzentrums (NRZ) für gramnegative Krankenhauserreger. Epidemiol. Bull..

